# Multi-Modal Single-Cell Sequencing Identifies Cellular Immunophenotypes Associated With Juvenile Dermatomyositis Disease Activity

**DOI:** 10.3389/fimmu.2022.902232

**Published:** 2022-06-21

**Authors:** Jessica Neely, George Hartoularos, Daniel Bunis, Yang Sun, David Lee, Susan Kim, Chun Jimmie Ye, Marina Sirota

**Affiliations:** ^1^ Division of Pediatric Rheumatology, Department of Pediatrics, University of California San Francisco School of Medicine, San Francisco, CA, United States; ^2^ Graduate Program in Biological and Medical Informatics, University of California San Francisco, San Francisco, CA, United States; ^3^ Institute of Human Genetics, University of California San Francisco, San Francisco, CA, United States; ^4^ Division of Rheumatology, Department of Medicine, University of California San Francisco, San Francisco, CA, United States; ^5^ UCSF CoLabs, University of California San Francisco, San Francisco, CA, United States; ^6^ ImmunoX Initiative, University of California San Francisco, San Francisco, CA, United States; ^7^ Department of Epidemiology and Biostatistics, University of California San Francisco, San Francisco, United States; ^8^ Bakar Computational Health Sciences Institute, University of California San Francisco, San Francisco, CA, United States; ^9^ Parker Institute for Cancer Immunotherapy, San Francisco, CA, United States; ^10^ Chan Zuckerberg Biohub, San Francisco, CA, United States; ^11^ Department of Pediatrics, University of California San Francisco, San Francisco, CA, United States

**Keywords:** juvenile dermatomyositis (JDM), dermatomyositis, autoimmune disease, single-cell sequencing, immune phenotyping, precision medicine, omics

## Abstract

Juvenile dermatomyositis (JDM) is a rare autoimmune condition with insufficient biomarkers and treatments, in part, due to incomplete knowledge of the cell types mediating disease. We investigated immunophenotypes and cell-specific genes associated with disease activity using multiplexed RNA and protein single-cell sequencing applied to PBMCs from 4 treatment-naïve JDM (TN-JDM) subjects at baseline, 2, 4, and 6 months post-treatment and 4 subjects with inactive disease on treatment. Analysis of 55,564 cells revealed separate clustering of TN-JDM cells within monocyte, NK, CD8^+^ effector T and naïve B populations. The proportion of CD16^+^ monocytes was reduced in TN-JDM, and naïve B cells and CD4^+^ Tregs were expanded. Cell-type differential gene expression analysis and hierarchical clustering identified a pan-cell-type IFN gene signature over-expressed in TN-JDM in all cell types and correlated with disease activity most strongly in cytotoxic cell types. TN-JDM CD16^+^ monocytes expressed the highest IFN gene score and were highly skewed toward an inflammatory and antigen-presenting phenotype at both the transcriptomic and proteomic levels. A transitional B cell population with a distinct transcriptomic signature was expanded in TN-JDM and characterized by higher CD24 and CD5 proteins and less CD39, an immunoregulatory protein. This data provides new insights into JDM immune dysregulation at cellular resolution and serves as a novel resource for myositis investigators.

## Introduction

Juvenile dermatomyositis (JDM) is a complex immune-mediated disease characterized by inflammatory myopathy of proximal musculature and pathognomonic skin rashes. Immune mechanisms are not fully understood, however, investigations to date implicate a combination of genetic variants and environmental influences (1). Though mortality rates have greatly improved, more than 60% of children with JDM have long-term damage and functional impairment due to poorly controlled disease or corticosteroid toxicity (2). This is, in part, due to the paucity of reliable, objective biomarkers to monitor disease activity, predict flares, or guide treatment decisions and an incomplete understanding of the cellular immunophenotypes contributing to disease. To improve the outcomes of children with JDM, a personalized approach to disease management, is urgently needed.

A key step in developing a personalized treatment strategy in JDM is a better understanding of the interferon (IFN) response, including the specific cell types involved in IFN signaling in JDM and the dynamic nature of the response during the course of disease. Numerous studies investigating gene expression in adult dermatomyositis (DM) and JDM have shown that the transcriptional signature is dominated by type I IFN stimulated genes (ISGs) (3–6). Some, but not all studies, have shown a correlation with ISG expression and disease activity (3, 7, 8). This discrepancy is likely due to the limitations of prior expression profiling technology, which measure transcriptional changes across aggregated cell types, as well as nuances of IFN signaling, which have been shown to be both cell-type-specific and disease-specific (9). Single-cell sequencing, using a multi-modal approach measuring gene and protein expression, represents a unique opportunity to characterize immune cell types and cell-specific IFN responses in JDM, in an unbiased fashion.

In addition to characterizing the cell-type specific IFN response in JDM, a better understanding of the cell types that contribute to disease is imperative. Immune mechanisms in JDM are complex, involving both innate and adaptive arms of the immune system, as well as many cell types (1). A role for B cells has been supported by the presence of myositis-specific antibodies that correlate with distinct clinical phenotypes (10) and by a positive clinical response to rituximab, a B-cell depleting agent (11). Likewise, two independent studies have also demonstrated an expansion of naïve B cells in JDM (12, 13). Several studies have demonstrated infiltration of T cells in muscle and skin biopsies in DM/JDM using immunohistochemistry (14–17), and flow cytometry studies demonstrated a skewing of peripheral blood CXCR5^+^Th subsets toward Th2 and Th17 phenotypes with increasing disease activity (18). Additionally, peripheral blood NK cells are decreased in number and dysfunctional, suggesting a role for innate immune cells as well (13).

In this study, we sought to build upon this knowledge by comprehensively interrogating the peripheral blood compartment in JDM, including simultaneous measurements of RNA and cell-surface proteins. Our goal was to provide an unbiased characterization of peripheral blood immune cells as well as insight into the dynamic nature of immune cell composition and cell-specific transcriptomic and proteomic signatures over time. An improved understanding of the immune cell types associated with JDM disease activity is integral to future biomarker and drug target development as we work to develop personalized medicine approaches to the care of JDM.

## Methods

### Patients

This study was approved by the University of California, San Francisco (UCSF) Institutional Review Board. Subjects meeting Bohan and Peter criteria for JDM (19), modified to include MRI as a possible diagnostic modality reflective of current practice, were recruited from the UCSF Pediatric Rheumatology Clinics in San Francisco and Oakland. All subjects provided informed consent, as well as informed assent, when age-appropriate. Patients could be enrolled at any time in their disease course. At each study visit, patient clinical data, which included items contained in the IMACS core consensus data set (20), was collected in a secure REDCap database. Patients enrolled at diagnosis also had follow up study visits at approximately 2, 4 and 6 months into treatment. For this study, inactive disease was defined using a modified PRINTO criteria for inactive disease (21): creatinine kinase <=150, manual muscle testing (MMT)-8<=78, and physician global visual analog score (VAS) <=0.5 as well as clinical judgement of inactive disease. We used a threshold of <=0.5 rather than 0.2 because our data collection forms included checked boxes in increments of 0.5 for VAS scores, and we did not collect CMAS measurements. Demographics, disease characteristics, and median disease activity measures for each patient group are summarized in **Table 1**. These measures are displayed graphically in **Supplementary Figure 1**.

**Table 1:** Patient Characteristics.

	I-JDM	TN-JDM			
		Baseline	2 months	4 months	6 months
**Median age (yrs) at diagnosis**	7	9.5	–	–	–
**Median age (yrs) at study visit**	9.5	9.5	-	-	-
**Female sex**	75%	50%	–	–	–
**TIF1-y (%)**	50%	50%	-	-	-
**NXP-2 (%)**	25%	25%	–	–	–
**MDA-5 (%)**	-	25%	-	-	-
**MSA-neg (%)**	25%	–	–	–	–
**Median disease duration (mos)**	38.2	2.7	-	-	-
**Physician VAS Global (median [IQR])**	0.4 [0.2, 0.5]	5.5 [4.6, 6.0]	2.3 [1.6, 2.9]	1.3 [0.8, 1.6]	1.0 [0.4, 1.5]
**Physician VAS Cutaneous (median [IQR])**	0.3 [0.0, 0.5]	4.5 [3.1, 5.9]	2.3 [1.6, 2.6]	1.8 [1.3, 2.0]	1.3 [0.8, 1.6]
**Physician VAS Muscle (median [IQR])**	0.2 [0.0, 0.5]	4.0 [3.3, 4.0]	0.8 [0.4, 1.0]	0.0 [0.0, 0.1]	0.0 [0.0, 0.1]
**MMT-8 (median [IQR])**	79.0 [78.0, 80.0]	70.0 [69.5, 73.0]	80.0 [78.5, 80.0]	80.0 [79.0, 80.0]	80.0 [79.5, 80.0]
**CHAQ Disability Index (median [IQR])**	0.00 [0.00, 0.09]	0.62 [0.62, 1.25]	0.31 [0.22, 0.69]	0.31 [0.09, 0.56]	0.06 [0.00, 0.19]
**CDASI Activity Score (median [IQR])**	1.0 [0.0, 2.3]	21.5 [13.0, 29.0]	5.5 [3.3, 7.8]	4.5 [3.3, 5.0]	3.0 [1.8, 4.3]

I-JDM, inactive juvenile dermatomyositis; TN-JDM, treatment-naïve juvenile dermatomyositis; MSA-neg, myositis-specific antibody negative; VAS, visual analog scale; IQR, interquartile range; MMT-8 manual muscle testing 8; CHAQ, childhood health assessment questionairre; CDASI cutaneous dermatomyositis disease area and severity index.

### Sample Processing & Multi-Modal Single-Cell Sequencing

Peripheral blood samples were collected at each study visit and processed by the Pediatric Clinical Research Core Sample Processing Lab. Peripheral blood mononuclear cells (PBMCs) were collected in SepMate tubes (n=5) or CPT tubes (n=15), isolated per each manufacturer’s guidelines, and cryopreserved in liquid nitrogen. Our experimental protocol followed the manufacturer’s user guide (10X 3’V3 Document CG000185 Rev B, 10X Genomics) with certain modifications to isolate and amplify antibody-derived DNA tags (ADTs). Note these experiments were carried out using early access kits from BD Genomics before the implementation of commercially-available single-cell protein/RNA assays (e.g. Feature Barcoding, 10x Genomics; BD Abseq, BD Genomics, **Supplementary Table 1**), and researchers are recommended to use those newer solutions for any follow-up studies as the techniques and reagents have been refined. For the experiment, PBMCs from 20 distinct samples were gently thawed in a 37°C water bath and re-suspended using a pipette set to 1 mL. Cell counts and viability were determined using a Cellometer Vision (Nexcelcom) with AOPI staining (Nexcelcom cat. CS2-0106-5ML). Cells were multiplexed into four pools with five samples each (2x10^5^ cells/sample) into separate 5 ml tubes (Falcon cat. 342235). Longitudinal samples from the same individual were aliquoted to distinct pools to enable genetic demultiplexing and experimental time points were also mixed within each well to avoid confounding time-related and batch effects. After pooling, cells were resuspended in 90 μl of 1% BSA in PBS and Fc blocked with 10 μl Human Trustain FcX (Biolegend cat. 422302) for 10 minutes on ice then stained on ice for 45 minutes with a pool of 50 antibodies in 100 μl, for a final staining volume of 200 μl. Antibodies were pooled on ice with 2.2 μl per antibody per 1x10^6^ cells (BD Genomics). Cells were quenched with 2 ml 1% BSA in PBS and spun at 350xg for 5 minutes and further washed two more times with 2 ml of 1% BSA in PBS. After the final wash, cells were resuspended in 100 ul and strained through a 40 μM filter (SP Bel-Art cat. H13680-0040). Each pool was loaded and processed into a respective well (4 wells total, 4x10^4^ cells/well) as in the manufacturer’s protocol. The 10x instrument was run and post-GEM RT and cleanup were done as according to manufacturer’s protocol. Starting at cDNA amplification, modifications to the protocol were made: 1 μl of 2 μM additive primer (BD Genomics, beta kit) specific to the antibodies tags was added to the amplification mixture. During the 0.6X SPRIselect (Beckman Coulter, B23318) isolation of the post-cDNA amplification reaction cleanup, the supernatant fraction was retained for ADT library generation. Subsequent library preparation of the cDNA SPRI-select pellet was done exactly according to protocol, using unique SI PCR Primers (10x Genomics). For the ADT supernatant fraction, a 1.8X SPRI was done to isolate ADTs from other non-specifically amplified sequences, followed by a sample index PCR. Sample index PCR for the ADTs was done using the cycling conditions as outlined in the standard protocol (15 cycles) but using different unique SI-PCR Primers such that all libraries could be mixed and sequenced together. Subsequent SPRI selection was performed, and all libraries were quantified and analyzed *via* Qubit 2.0 (Fisher) and Bioanalyzer (Agilent), respectively, for quality control. Libraries were mixed and sequenced on 1 lane of a NovaSeq S4 using the recommended number of cycles.

### Alignment, Demultiplexing, and Doublet Removal

CellRanger (v3.1.0) was run to align reads to the GRCh38 genome build and generate counts matrices and binary alignment files (BAMs). Each BAM was inputted into freemuxlet for donor-of-origin annotation and doublet removal. To assign cells to donors of origin in our multiplexed design, we used the genetic demultiplexing tool freemuxlet and a sample matching script, each being part of the popscle suite of population genetics tools (https://github.com/statgen/popscle) to assign cells to donors as previously described (22). To create the external genotype reference, DNA was extracted from whole blood samples for each patient and genotyped using the OmniExpressExome array at the UC Berkeley Vincent J. Coates Genomics Sequencing Laboratory. Freemuxlet and the sample matching script were run, yielding a 1 to 1 mapping of droplet barcode clusters to individuals. Heterotypic doublets detected by demuxlet were removed, and additional homotypic doublets (i.e. two cells from the same individual co-encapsulated) were removed using doubletdetector (23).

### Quality Control and Processing

Gene and protein expression matrices (4 each) were subsequently processed using Scanpy (v1.5.1) (24). To identify and filter low quality cells, we visualized the log-normalized distributions of mRNA counts, protein counts, and gene counts for each of the four wells and filtered counts at the tails of these distributions. The percent mitochondrial gene expression for each well displayed a similar distribution, so we applied the same cutoff of filtering cells with >15% mitochondrial gene expression to all four wells. After filtering and doublet removal, we analyzed 55,564 cells. The average number of cells sequenced per sample was 2,778. Subject N-4 had significantly few cells sequenced than the three other newly diagnosed patients (n=2,663), compared to N-1 (n=13,812), N-2 (n=11,985), N-3 (n=11,633)), particularly for visits 2 and 3 where only 12 and 116 cells were recovered, respectively. This was taken into account for all downstream analyses. A similar number of cells, ~14,000 was recovered from each of the four 10X wells.

The four mRNA matrices were then concatenated and log-normalized. We then extracted a set of highly-variable genes using the Scanpy function “highly_variable_genes” and then selecting a set of genes with high mean expression and dispersion. The protein matrices were also concatenated and log-normalized, and the both RNA and protein matrices were merged for down-stream join clustering. The Scanpy commands “regress_out”, to control for the effect of mitochondria gene count and total mRNA count, and “combat” (25), to control the batch effect of the four wells, were used. Batch correction using ComBat was able to correct for technical replicates except in CD14^+^ monocytes, where there appears to be some residual sample-specific effects especially related to cluster 18. These effects could be due to batch or other covariates such as sex, which is consistent with previous literature showing significant inter-individual variation in monocytes (26, 27) (**Supplementary Figure 2**). We accounted for these effects by analyzing these clusters collectively in down-stream analysese. Clusters were otherwise independent of patient effect, however, there was some patient-level heterogeneity: N-2 cells make up a large majority of CD8^+^ effector T cells and N-1 and N-3, both males, appear to cluster together within the CD14^+^ monocyte population (**Supplementary Figure 3**). Principal component analysis was applied to identify 30 PCs, and the “neighbors” command was used to compute a neighborhood graph using the default size of 15. We then embedded the neighborhood graph using Uniform Manifold Approximation and Projection (UMAP) (28) for subsequent visualization using the default settings.

### Clustering and Cell Type Annotation

Clustering was then performed using the set of highly variable genes and all proteins and applying the Leiden algorithm (29) with a resolution of 1.5, which identified 24 cell clusters, 23 of which were immune cell clusters (cluster 22 was platelets), including all major immune cell populations (**Supplementary Figure 4**; **Figure 2A**). Two clusters, 13 and 15, could not be annotated and expressed both CD8 and mRNA transcripts for the gamma and delta chains of the T cell receptor. These two clusters were further subclustered at a low resolution using the “restrict_to” parameter in the Leiden function in Scanpy to identify distinct CD8^+^ memory T cell and gamma-delta T cell populations. Cells were annotated using canonical RNA and cell surface markers (**Figure 2A**; **Supplementary Figure 5**).

### Cell Type Compositional Analysis

To determine cell types enriched in treatment-naïve disease, cell type proportion was compared between the baseline and 6-month visit using a paired t-test and significance threshold of p<0.05. We also compared cell type proportion between TN-JDM, baseline visit, and inactive JDM (I-JDM) using a t-test and a significance threshold of p<0.05. For both analyses, percent composition was calculated and visualized with dittoSeq’s “dittoBarPlot” function (30).

### Differential Gene and Protein Expression

To calculate cell type differential gene and protein expression between disease states, we used the DESeq2 package (v1.28.1) (31), using the inputs recommended for single-cell data in the package vignette (test = “LRT”, reduced = ~1, sfType = “poscounts”, useT = TRUE, minmu = 1e-6, minReplicatesForReplace = Inf) and included batch as a covariate in the design formula. We used a log-fold change cutoff of >1 for differential gene expression or >0.5 for protein expression, and a false discovery rate <0.05. Only genes and proteins expressed in at least 10% of cells for each cell populations were assessed. Differential expression was not calculated for plasmablasts, due to low cell numbers, or platelets.

### Global Cell-Specific Transcriptional Signature

To identify and visualize the global cell-specific transcriptional signature, we collated the set of non Y-chromosome genes that were differentially expressed in at least one cell type in both analyses longitudinal and cross-sectional analyses. The pseudobulk mean expression per sample group, defined as baseline, 2, 4, and 6 months and inactive, for each of the genes per cell type was calculated and visualized using dittoSeq’s ‘dittoHeatmap’ (30). Columns were ordered by cell type and group by increasing time from diagnosis, which also correlated with decreasing disease activity levels. Unsupervised hierarchical clustering by Euclidean distance was then applied to cluster the genes into distinct modules using k=10. Module scores were then calculated as the sum of mean expression of all module genes for each pseudobulk calculation per sample group. Correlations of module scores (minimum of 10 cells per case) to X-metric was then calculated with using the Pearson correlation and visualized as dot & scatter plots using ggplot2 (32). Gene Ontology enrichment analysis was applied to each gene set using clusterProfiler (33) over-representation analysis and Gene Ontology Biological Processes reference and a significance cut-off of p<0.05, Benjamini-Hochberg (B&H) adjusted.

### Identifying Cell Type Subclusters

To identify subclusters within selected cell populations, a second round of clustering was applied using the “restrict_to” parameter in the Leiden function in Scanpy and a low clustering resolution of 0.2 for CD16^+^ monocytes and 0.3 for naïve B cells. The proportion of cells from each sample per subcluster was visualized using barplots. The proportion of cells from each patient group was calculated and a chi-square test was applied to determine if the composition of subclusters differed. Differential gene expression and differential protein expression for each subcluster compared to the canonical cell population was calculated using DESeq2 in the same method as described above.

## Results

### JDM Is Associated With Alterations in Immune Cell Composition

**Figure 1:**
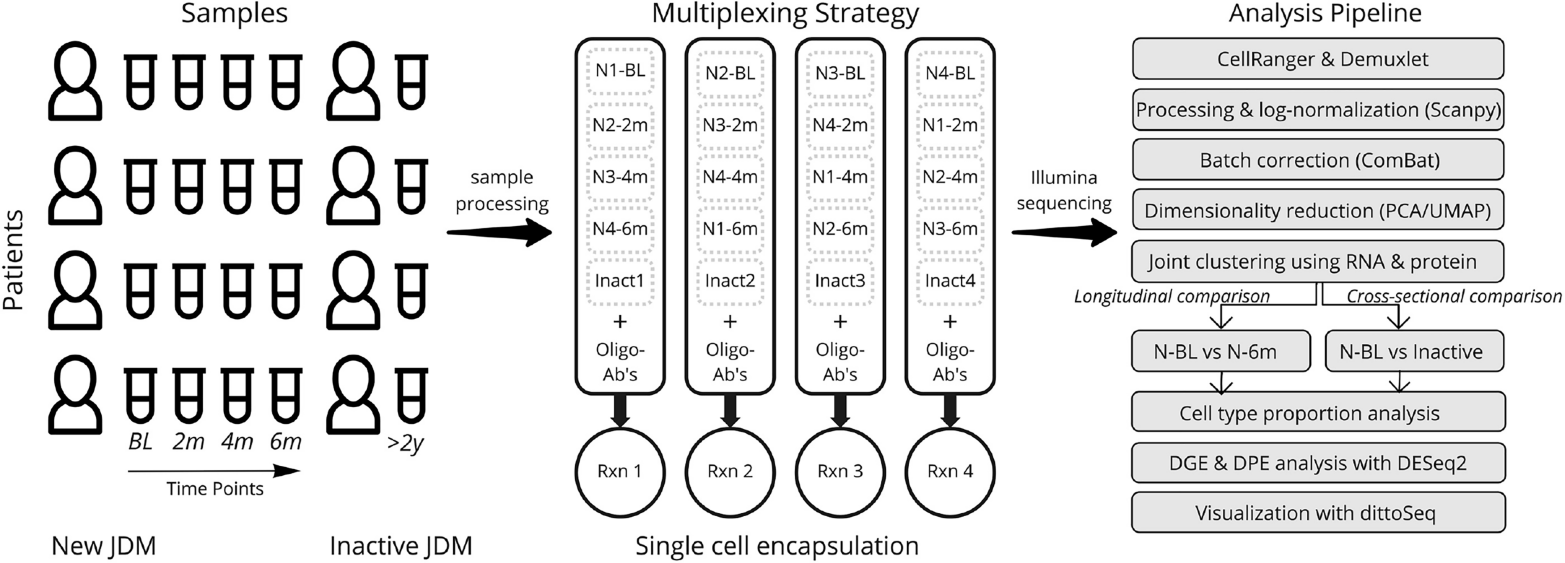
An overview of the experimental design, multiplexing strategy, and analysis pipeline. Timepoints refer to longitudinal samples obtained from the same patients at BL=baseline, 2m=2 months, 4m=4 months, 6m=6 months; N refers to “New-JDM”. Rxn, reaction; DGE, differential gene expression; DPE, differential protein expression.

A graphical depiction of our study design, multiplexing strategy, and analysis pipeline is depicted in **Figure 1**. A total of 20 samples from 4 subjects with JDM with treatment-naïve disease (TN-JDM) and 4 subjects with established, clinically inactive disease (I-JDM) were included. Serial samples from TN-JDM subjects were included at baseline and approximately, 2, 4, and 6 months into treatment. The median age of JDM diagnosis was 9.5 years in the TN-JDM group and 7 years in the I-JDM group, and the median age at study enrollment in both groups was 9.5 years (**Table 1**). Two of four patients were female in the TN-JDM group and 3 out of 4 were female in the I-JDM group. In both groups, 2/4 patients were positive for TIF1-γ. All patients exhibited improvement in disease activity measures during the first 6 months of disease (**Table 1** and **Supplementary Figure 1**).

**Figure 2:**
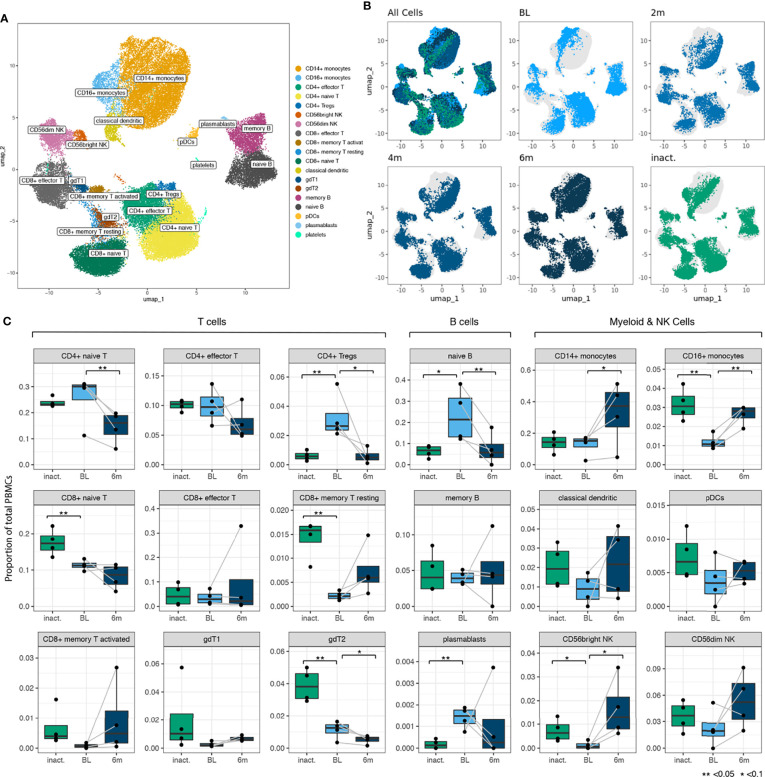
**(A)** A UMAP plot showing 19 manually annotated cell types from 24 immune cell populations identified by Leiden clustering. Manual annotations were based on canonical markers shown in **Supplementary Figure 5**. **(B)** UMAP embeddings showing all cells colored by timepoint (for newly-diagnosed patients) and inactive disease state showing distinct regions of embedding for cells from baseline (BL) TN (treatment-naïve) patients in most cell populations. **(C)** Boxplots for 18 cell types (platelets not included) showing differences in cell type proportion between BL TN samples and inactive samples and between BL, TN samples and 6m samples. Comparison of proportions between BL and 6 months was a paired analysis and grey lines connect the observations from the same individuals. P values were calculated using a T test (**<0.05, *<0.1).

After filtering and doublet removal, we analyzed 55,564 cells comprising all the major immune cell populations, which were annotated using canonical cell markers (**Figure 2A**). Cells from TN-JDM subjects clustered together and occupied distinct regions of UMAP embeddings in CD14^+^ and CD16^+^ monocyte, naïve B cell, naïve and effector CD4^+^ and CD8^+^ T cell and CD56^dim^ NK cell populations (**Figure 2B**). Visualization of UMAP embeddings by visit demonstrated a shift in embeddings over time from tightly clustered regions in TN-DM to a broader embedding across the cluster at subsequent time points suggesting diversification of cell states as disease activity declines with treatment (**Figure 2B**).

Cell type proportions were altered in TN-JDM compared to treated JDM (**Figure 2C**). In a paired analysis of TN-JDM comparing baseline samples to 6 month samples, there was a significant expansion of naïve B cells (p=0.03) and CD4^+^ naïve T cells (p=0.02) and a reduction of CD16^+^ monocytes (p=0.01) in TN-JDM. One patient did not have any B cells at the 6 months time point due to treatment with a B-cell depleting agent, rituximab. In a cross-sectional analyses comparing TN-JDM to I-JDM, there was also a reduction of CD16^+^ monocytes (p=0.01) and trend toward expansion of naïve B cells (p=0.07) in TN-JDM. There were also significant differences in several T cell populations with expansion of CD4^+^ Tregs (p=0.04) and a reduction of CD8^+^ naïve T cells (p=0.04), and gdT2 populations (p=0.01) in TN-JDM. The increase in the CD8^+^ memory T resting (p=0.01) population in inactive JDM relative to TN-JDM suggests a memory T cell response that develops over time in JDM. Expansion of naïve B cells, and alterations in regulatory cell types, including CD16^+^ monocytes and CD4^+^Tregs, may be associated with TN-JDM.

### Global and Cell Type-Specific Transcriptomic Signatures in JDM Are Associated With Disease Activity

**Figure 3:**
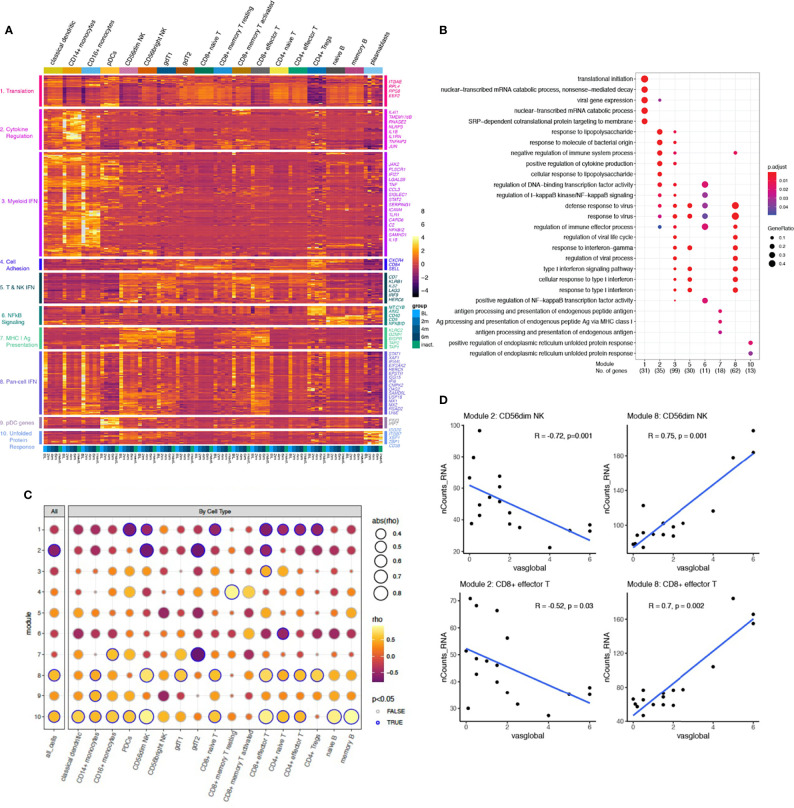
**(A)** A heatmap displaying the mean expression of 368 genes differentially expressed in at least one cell type for each group per cell type ordered by increasing time from diagnosis (BL, 2m, 4m, 6m, inactive). Rows are clustered using unsupervised hierarchical clustering with k=10, and modules are annotated by enrichment terms on the left. A selection of genes from each module is annotated on the right. Color represents the normalized mean expression. **(B)** A dotplot displaying the association between each gene module set and enrichment of GO Biologic Processes terms. Each column represents a module from panel **(A)**. Enrichment analysis was performed using over-representation analysis with the color representing the adjusted p-value using B&H and the size of the dot representing the ratio of the number of genes relative to the number of genes in each term. **(C)** A dotplot displaying the strength of correlation between each module gene score, calculated as the average expression of all genes within a module, and the physician global VAS score for all cells together (left column) and each cell type individually. The correlations were calculated using a Pearson correlation. The size of each dot represents the strength of the association, the color represents the direction of the association, and the outline of the circle indicates significance defined as P-value <0.05. **(D)** The Pearson correlation between the module gene score and physician global VAS plotted for each sample in CD8^+^ effector T and CD56^dim^ NK cells for modules 2 and 8. The blue line represents a linear model fit for visualization purposes.

To develop a global understanding of the JDM transcriptional signature and how it changes with treatment, we first performed differential gene expression analysis between TN-JDM baseline and 6 months visits and between TN-JDM and I-JDM groups (**Supplementary Table 2**). Monocytes, classical dendritic cells (cDCs), and cytotoxic cell types, CD8^+^ effector T and CD56^dim^ NK cells, displayed the greatest number of differentially expressed genes (DEGs) and many genes were differentially expressed in multiple cell types (**Supplementary Figures 6**, **7**). Unsupervised clustering using cell-specific DEGs clustered patients according to disease activity levels, as expected (**Supplementary Figures 8**–**13**). We then calculated the pseudobulk expression profiles per disease group (baseline, 2 mos, 4 mos, 6 mos, and inactive) for 368 genes that were differentially expressed in at least one cell type in both analyses (**Figure 3A**). Hierarchical clustering of this gene set revealed 10 gene modules, which were visualized using a heatmap with columns ordered by descending level of disease activity (baseline, 2 mos, 4 mos, 6 mos, and inactive) and cell type. Gene ontology enrichment analysis of each of the ten gene modules identified associations with protein translation, cytokine regulation, type I and II interferon signaling, NFκB signaling, antigen presentation *via* the class I pathway, and the unfolded protein response (**Figure 3B** and **Supplementary Figure 14**). Module 9 did not have any significant enrichment terms, but these genes were highly expressed in plasmacytoid dendritic cells (pDCs). Three modules were enriched in IFN signaling, including type I and type II IFN responses: modules 3, 5, and 8 (**Figure 3B**). Module 8 genes were highly expressed in the TN-JDM group in nearly every cell type, and expression sharply declined with treatment. This “Pan-cell IFN” signature was most prominently expressed in CD16^+^ and CD14^+^ monocytes, CD56^dim^ NK cells and CD8^+^ effector T cells in TN-JDM subjects (**Supplementary Figure 15**). In contrast, module 3 genes were more prominently expressed in myeloid cell types and module 5 genes were more prominently expressed in T and NK cell types, and gene expression within these cell types displayed a more static expression pattern over time for the respective IFN gene modules. CD16^+^ monocytes displayed the highest gene scores in TN-JDM patients for both the myeloid IFN module and the “Pan-cell IFN” module (**Supplementary Figure 15**).

We next wanted to directly determine the association between these gene sets and disease activity measures to identify biologically relevant sets of genes and putative disease activity biomarkers. We calculated a gene score for each module by averaging the expression of the gene set for each sample. The Pearson correlations between the gene score per sample and physician global visual analog score (VAS) score identified a significant correlation in bulk cells for modules 2, 8 and 10 (**Figure 3C**). Evaluation of the correlations between cell-specific gene scores and disease activity identified stronger correlations in CD56^dim^ NK and CD8 effector T cells for all three modules (**Figures 3C, D**). These cell-specific gene scores were negatively associated with disease activity for module 2 genes and positively correlated with module 8 genes (**Figure 3D**), which may be suggestive of opposing gene programs that become more regulated with treatment. The magnitude of change in gene scores was greatest within the module 8, “Pan-cell IFN” gene set. Module 10 gene scores displayed very strong correlations in memory and naïve B cells, though the overall magnitude of the change was less. This gene set was also highly expressed in plasmablasts (**Figure 3A**). These results help to identify ISG’s expressed more dynamically in JDM from those that are more intrinsically expressed in in certain cell types and suggests that the pan-cell IFN gene signature might serve as a disease biomarker in JDM in cytotoxic cell types and in bulk cells.

### Monocytes in TN-JDM Display Inflammatory and Antigen-Presenting Properties

**Figure 4:**
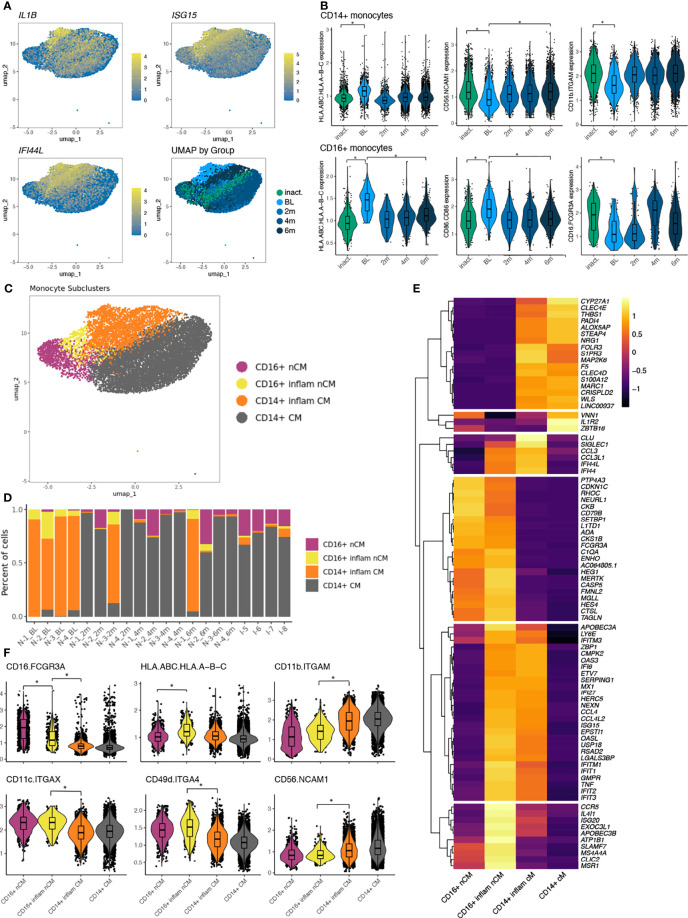
**(A)** UMAP plots of the monocyte population colored by select features where color represents the log-normalized expression. The fourth panel is colored by disease group. **(B)** The normalized expression of differentially expressed protein markers in baseline (BL) samples compared to inactive (inact.) samples as well as to 6 month (6m) samples. Significance is indicated by a log-fold change >0.5 and FDR<0.05 indicated by *. **(C)** UMAP of monocyte populations colored by monocyte cell states following a second round of clustering of CD16^+^ nCM. **(D)** The proportion of cells making up the monocyte subclusters per sample ordered by time and disease group. **(E)** A heatmap displaying differentially expressed transcripts between monocyte populations using a log-fold change >3 and adjusted p-value <0.05. All genes differentially expressed meeting significance thresholds are displayed in **Supplementary Table 3**. **(F)** Violin plots displaying expression values of differentially expressed proteins between monocyte subsets using a log-fold change >0.5 and adjusted p-value <0.05 indicated by *.

We next turned our attention to the monocyte populations because of the strong IFN signature, sizeable number of DEGs, and compositional changes observed between disease groups. Differential gene expression results were concordant comparing TN-JDM baseline samples to 6 months samples and TN-JDM to I-JDM in both cell types. TN-JDM subjects displayed up-regulation of IFN-stimulated genes and inflammatory genes, including *NFKBIZ, NFKBIA, IL1B, TNF, CCL3*, and *CCL4* in both monocyte populations (**Supplementary Table 2**). Despite a reduction in CD16^+^ monocytes in TN-JDM, this population had the most differentially expressed genes both comparing TN-JDM baseline to 6 mos (n=229) and TN-JDM baseline to I-JDM (n=359) (**Supplementary Figures 6**, **7**). In addition to ISG’s and inflammatory cytokines, TN-JDM subjects also displayed up-regulation of genes related to antigen presentation (*CD40, HLA.DRB5, HLA.DRB1*, and *TAPBL*), TLR signaling (*TLR1* and *TLR2*), inflammasome signaling (*NLRP3* and *AIM2*), IL-6 signaling (*IL6ST*) and complement genes (*C2, C1QA*), indicating a population with inflammatory and antigen-presenting capabilities. Within the CD14^+^ monocyte population, TN-JDM subjects also displayed less expression of *CD163* and *IL-18* compared to treated JDM suggesting reduced phagocytic function of the CD14^+^ monocyte population in active disease. In both monocyte populations, we observed co-expression of genes related to IL-1 signaling and IFN signaling (**Figure 4A**) that corresponded to the region of embedding of TN-JDM cells (**Figure 4A**). Differential protein analysis within CD14^+^ monocytes revealed up-regulation of HLA.A-B-C, a marker for MHC class I, in TN-JDM and down-regulation of CD56 and CD11b (**Figure 4B**). In CD16^+^ monocytes, there was up-regulation of HLA.A-B-C and CD86, a costimulatory molecule, supporting the antigen-presenting capabilities of these cells at the protein level (**Figure 4B**). Notably, we observed down-regulation of the surface CD16 in TN-JDM cells (**Figure 4B**).

Because of the high number of DEGs and because TN-JDM cells occupied a distinct region within monocytes in the UMAP, we next evaluated the role of monocyte subsets. A second round of clustering was applied to the CD16^+^ monocyte population and identified a cluster consisting of 98% TN-JDM CD16^+^ cells (**Figure 4C**). The composition of this subcluster was significantly different with a high proportion of cells from TN-JDM (**Figure 4D**; **Supplementary Figure 16**). Likewise, the majority of TN-JDM cells in the CD14^+^ monocyte population resided within original Leiden clusters “10” and “18”, which we termed CD14^+^ inflammatory monocytes (**Figure 4D**; **Supplementary Figure 16**). Both of these subclusters expressed high levels of inflammatory genes like *CCL3, CCL4, TNF* and ISG’s but also retained features of the original CD16^+^ and CD14^+^ monocytes, supporting inflammatory cell states of CD16^+^ and CD14^+^ monocytes in TN-JDM (**Figures 4E, F**). At the gene level CD16^+^ inflammatory monocytes displayed higher expression of *CCR5* (not recapitulated at the protein level), *IL4IL*, and *ATP1B* than other monocyte subclusters (**Figure 4E**) and differential expression of many HLA class II genes and CD14^+^ inflammatory monocytes displayed more S100 protein genes, including *S100A8, S100A9*, and *S100A12* (**Supplementary Table 3**). At the protein level, CD16^+^ inflammatory monocytes displayed less CD16^+^ expression and distinguished from CD14^+^ monocytes by increased expression of, CD49d, CD16, CD11c and reduced CD11b and CD56 (**Figure 4F**). While this population displayed less CD16^+^, *CD14* transcripts were not significantly different between the two CD16^+^ clusters, and additional canonical markers of intermediate monocytes were not present supporting the classification of this cell type as a CD16^+^ monocyte population exhibiting an altered inflammatory cell state.

The transcriptomic and proteomic features exhibited by these monocyte populations is suggestive of early macrophage differentiation. Interestingly, both CD16^+^ and CD14^+^ inflammatory monocytes display features resembling pro-inflammatory M1 macrophages; CD14^+^ monocytes also display some features resembling anti-inflammatory M2 macrophages, like *VEGFA* and *IL1R2* (decoy receptor) (**Supplementary Table 3**). These results support a role for the peripheral blood monocyte compartment in JDM, potentially as precursors to monocyte-derived macrophages in target tissues.

### A Transitional B Cell Population Is Expanded in TN-JDM

Two populations of B cells (CD19+, CD20+) were identified: naïve (IgD+) and memory B cells (CD27+), as well as a small cluster of plasmablasts (CD27+, CD38+). Naïve B cells were consistently expanded in TN-JDM both longitudinally comparing the baseline visit to 6 month visit and compared to I-JDM. Differential gene expression analysis revealed increased ISG expression in TN-JDM both longitudinally and compared to I-JDM (**Supplementary Table 2**). Differential protein expression identified down-regulation of CD39 in TN-JDM in both naïve B cells and memory B cells (**Supplementary Table 2**).

**Figure 5:**
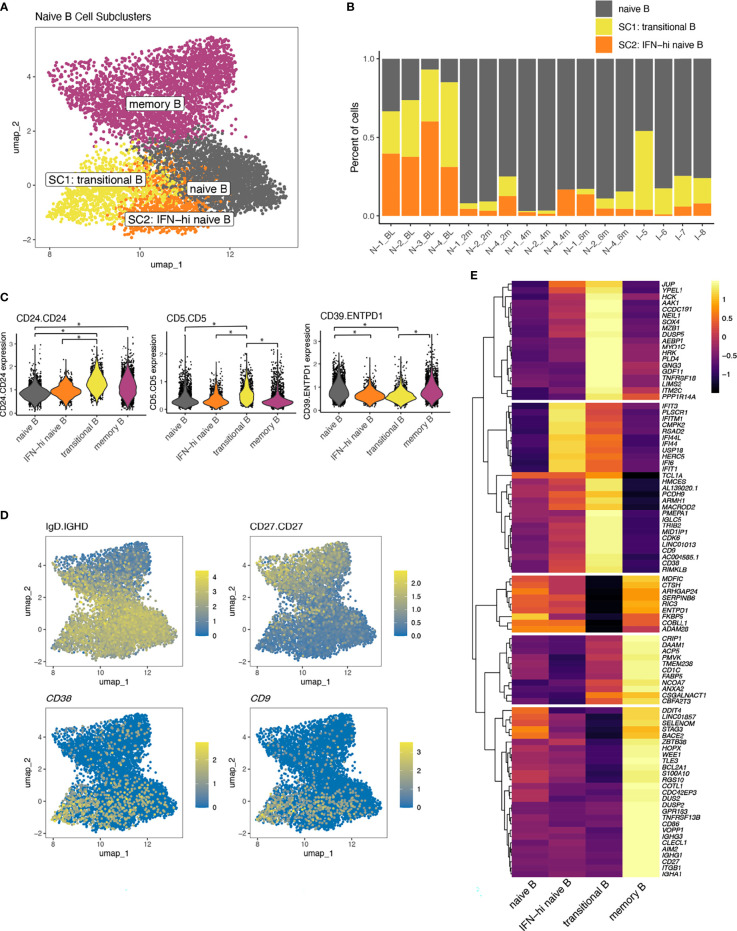
**(A)** A UMAP of B cell populations after a second round of naïve B cell clustering colored by naïve B cell subcluster. **(B)** The proportion of cells making up the naïve B cell subclusters per sample ordered by time and disease group. **(C)** Violin plots displaying expression values of differentially expressed proteins between naïve B cell subsets using a log-fold change >0.5 and adjusted p-value <0.05 indicated by *. **(D)** UMAPs of B cell populations displaying log-normalized expression of phenotypic markers IgD (protein), CD27 (protein), *CD38* (RNA) and *CD9* (RNA). **(E)** A heatmap displaying differentially expressed transcripts comparing transitional B cells to all other B cells and IFN-high naïve B cells to all other B cells. Genes displayed are those with a log-fold change>1.3 and adjusted p-value <0.05. All genes differentially expressed meeting log-fold change >1 are displayed in **Supplementary Table 4**.

Within the naïve-B cell population, cells from TN-JDM subjects occupied a distinct region (**Figure 2B**). To better characterize the cell types in this region, a second round of clustering was applied to the naïve B cell population, which identified two additional B cell subclusters (**Figure 5A**). These subclusters (SCs) displayed altered composition compared to the naïve B cell cluster with a high proportion of cells from TN-JDM subjects: 64% of SC1 and 87% of SC2 were from TN-JDM subjects (**Figure 5B**). Differential protein expression analysis comparing each cluster identified higher CD24 and CD5 expression and down-regulation of CD39 in SC1 (**Figure 5C**). SC2 was differentiated from naïve B cells only by down-regulation of CD39 (**Figure 5C**). SC1 also expressed surface IgD but not CD27 supporting the classification of this cell type as a transitional B cell population (34, 35) (**Figure 5D**).

Differential gene expression analysis comparing SC1 to all other B cells revealed a distinctive transcriptomic signature, including *CD38* and *CD9*, supporting classification as a transitional B cell population (**Figure 5D**), as well as many other genes not typically expressed at high levels in naïve B cells, including *TNFRSF18* (aka *GITR), PLD4, NEIL1*, and *ITM2C* (**Figure 5E**; **Supplementary Table 4**). SC1 displayed intermediate levels of ISG expression and notable down-regulation of several regulatory genes, including *ENTPD1*(**Figure 5E**), recapitulating differential protein results of CD39 down-regulation, *FCRL3* and *IL10RA* (**Supplementary Table 4**). SC2 was embedded between the transitional B cells and naïve B cells and was characterized by high ISG expression and intermediate levels of *CD38*, suggesting this population could also be a transitional population or a naïve B cell population in an altered IFN state, which we termed “IFN-hi naïve B” (**Figure 5E**). Transcriptomic expression of *ENTPD1* was not significantly different between IFN-hi naïve B and naïve B suggesting surface level expression of CD39 may be regulated post-transcriptionally in these naïve B cell populations. Together, the expansion of naïve B cells in the treatment-naïve state and the distinctive transcriptomic and proteomic properties of these naïve B cell subclusters, suggest a role for the B cell compartment in JDM.

## Discussion

This is the first comprehensive evaluation of the immunophenotypes associated with disease activity in the peripheral blood compartment in JDM using multi-modal single-cell sequencing. We identified changes in cellular composition, characterized cell-specific IFN responses, and identified unique cell immunophenotypes associated with treatment-naïve disease. We also show that these changes occur both within individuals and between individuals with JDM by including longitudinal samples. Furthermore, by measuring both gene expression and surface protein expression, we were able to identify multi-modal features associated with TN-JDM. Cell surface proteins are not only important for cell phenotyping but also for carrying out key cellular functions providing greater insight into cellular behavior. By including oligonucleotide-barcoded antibodies for 50 cell surface proteins, we identified surface markers, such as CD5 and CD39 in B cells, not typically associated with these immune cell types that displayed differential expression associated with disease activity in JDM. These results suggest that the peripheral blood compartment holds promise to help provide insights into disease mechanisms and to identify candidate biomarkers and therapeutic targets.

In accordance with the striking transcriptomic IFN signature previously described in JDM blood and target tissues (3–6), as expected, we also identified a strong IFN signature in our study. However, we did not anticipate that nearly every immune cell type would over-express a subset of IFN genes in the treatment-naïve state in our patient cohort. This pan-cell IFN rewiring strongly suggests that IFN signaling, either due to overactivation or lack of regulation, is a hallmark of JDM pathophysiology. CD16^+^ monocytes displayed the highest IFN expression, and other myeloid cell types (CD14^+^ monocytes and cDCs) and cytotoxic cell types (CD8^+^ T effector and CD56^dim^ NK) also displayed higher IFN gene scores than other cell types suggesting these are the primary peripheral blood mediators of the IFN response observed in JDM. Using single-cell sequencing, we were able to tease apart IFN-related genes expressed across bulk cells from genes expressed in a cell-type specific manner overcoming limitations of prior bulk sequencing experiments where expression is confounded by cell composition. The combination of the pan-cell and cell-specific IFN responses we observed in JDM may explain why studies correlating IFN gene signatures and disease activity have been mixed (3, 7). The pan-cell IFN signature we identified was correlated with individual disease activity measures in our small study in bulk cells and more strongly in CD8^+^ effector T and CD56^dim^ NK cells. Larger studies will be needed to determine the utility of this signature as a disease activity biomarker. This data, as well as prior literature (36, 37) would support the hypothesis that a carefully curated IFN gene signature correlates with disease activity in JDM.

Our data have identified the peripheral blood monocyte compartment to be highly dysregulated in JDM. In addition to exhibiting the strongest IFN response in TN subjects of all immune cell types and the greatest number of DEGs, CD16^+^ monocytes were quantitatively reduced in TN-JDM subject PBMCs and there was a trend toward reduction of CD14^+^ monocytes. We hypothesize this is due to tissue homing based on previous work from our group which identified strong enrichment of myeloid-derived cell types in muscle and skin microarray datasets (6) and recent cutting-edge work using mass cytometry imaging, which identified myeloid-derived cell types as the most abundant cell types in DM skin lesions (38). The role of CD16^+^ nonclassical monocytes in health and disease has been both controversial and context-dependent, but this cell type has traditionally been thought to be immune-regulatory (39). Prior work suggests CD16^+^ monocytes are predisposed to become migratory dendritic cells (40) or M2 anti-inflammatory macrophages (41). However, we identified a cluster of cells from TN-JDM subjects that were skewed toward an inflammatory and antigen-presenting phenotype more suggestive of pro-inflammatory M1 macrophages. This CD16^+^ inflammatory population also displayed down-regulation of CD16, the Fc receptor FcγRIIIA. This receptor is internalized upon the binding of immune complexes (42) leading us to hypothesize that active signaling through this receptor may be the mechanism of CD16 downregulation. This model would provide a possible link between autoreactive B cells, myositis-specific antibodies, and activation of CD16^+^ inflammatory monocytes, which subsequently migrate to tissues and differentiate into pro-inflammatory macrophages sustaining local inflammation. This model is also supported by evidence that suggests IVIG, an effective therapy in JDM, may work through blockade of activating Fc receptors (42). Alternatively, these cell types may be highly plastic and recruited to tissues to support tissue repair, or they may be detected in peripheral blood because they lack the appropriate tissue homing receptors. Further work to determine the trajectory of these cell types in target tissues is needed to determine the utility of targeting this cell type therapeutically.

There is also growing evidence to support a role for myeloid cell types, especially CD16^+^ monocytes, in other autoimmune diseases, including in systemic lupus erythematosus (SLE) (43), another IFN-mediated disease closely related to JDM. In TN-JDM, we observed a skewing of both CD16^+^ and CD14^+^ monocytes toward an inflammatory and antigen-presenting state, co-expressing IFN and IL-1 axis genes, consistent with a finding recently described in childhood lupus (44). This study also identified a correlation with the lupus IFN signature in CD16^+^ monocytes. A similar inflammatory CD16^+^ non-classical monocyte population has also been identified in adult SLE peripheral blood (43). These findings also extend into tissue where single cell sequencing of infiltrating immune cells in the kidneys of lupus nephritis patients also identified a continuum of CD16^+^ macrophage cell types, including inflammatory CD16^+^ macrophages without a CD14 counterpart (45). In adult dermatomyositis, peripheral blood monocytes display increased expression of TLR’s (46).

Different patterns of integrin and adhesion molecule surface expression distinguish monocyte populations in our study. CD11b and CD11c are well known markers distinguishing monocyte subsets, but we additionally observed CD49d as an integrin distinguishing CD16^+^ inflammatory monocytes from CD14^+^ inflammatory monocytes. There also appears to be a qualitative increase in CD49d in CD16^+^ inflammatory monocytes compared to non-inflammatory CD16^+^ monocytes, but we may have lacked power to detect a statistically significant difference for this marker between these two populations. CD49d is the alpha chain of the very late antigen 4 (VLA-4) integrin that binds to ligands fibronectin and VCAM-1 expressed on endothelium, the latter which was found to be up-regulated on muscle vessels in DM but not JDM (47). In Duchenne muscular dystrophy, a genetic myopathy with an inflammatory component, CD49d-expressing T cells displayed greater migratory responses and adhesion to myotubules, and inhibition of CD49d is being studied as a novel therapeutic option (48).

We identified expansion of a transitional B cell population with increased CD24 and CD5 expression as well as CD39 down-regulation and differential expression of several immunoregulatory genes, including up-regulation of *TNFRSF18 (GITR), PLD4*, and down-regulation of *ENTPD1, IL10RA*, and *FCRL3*. It is unclear whether this transitional population is a precursor to naïve B cells or an altered naïve B cell state in response to ISG stimulation. In this case, the ISG-hi naïve B cell population may represent a continuum of naïve B cell activation. Alternatively, these may represent two separate populations: one IFN-stimulated naïve B cell population and one transitional B cell population with distinct functional properties. Prior literature also identified an expansion of CD19+CD24hi, CD38hi transitional B cells in JDM and a correlation of the proportion of these cells with the type I IFN signature in B cells (12). It seems plausible that the transitional B cell population identified in this study is analogous providing further support that this cell type may be relevant to the disease process. We identify several additional features of this immunophenotype, including key genes and surface proteins, like CD5 expression.

The role of CD5 in naïve B cell populations has been controversial, described by some as an activation marker and by others as a distinct phenotypic B cell subset marker (49). In SLE, a CD5+ pre-naïve B cell population has been described that displayed functional properties of plasma cell differentiation and antigen-presentation, which the authors postulated to represent a mechanism of autoreactive B cell escape (50). We hypothesize that B cell development is altered due to the overactive IFN response in JDM, which may result in activation and proliferation of these transitional B cell populations, some of which may escape tolerance checkpoints. Furthermore, we observed, in both naïve and memory B cells, down-regulation of CD39 in TN-JDM and increased expression during the first 6 months of treatment during which all patients demonstrated a decrease in disease activity. The down-regulation of CD39 in these naïve B cell populations in TN patients may also contribute to such potential escape of autoreactive B cells from regulatory mechanisms like CD39-mediated immune regulation, a phenomenon also described in rheumatoid arthritis (51), or IL-10 production (12). CD39, or *ENTPD1*, contributes to adenosine production which has several anti-inflammatory effects. The role of this receptor is most well characterized in Tregs in human autoimmune disease, including SLE and RA, and is also of interest as a therapeutic target (52).

The expression of *TNFRSF18 (GITR)*, a co-stimulatory molecule, in JDM transitional B cells is of interest. It may simply represent a marker of transitional B cells with no functional consequences on B cell maturation, as suggested in murine studies (53), or represent an important signaling pathway by which these cells become dysregulated or influence lymphocytes. Expression of GITR ligand by B cells has been found to play an important role in the maintenance of Treg populations in mouse autoimmune models, but there is little to no data studying the role of GITR in transitional B cells in human disease states. Given the success of many immunotherapy agents, there is great interest in this receptor as a therapeutic target for both cancers and autoimmune diseases. Likewise, *PLD4* is also of interests as it is an exonuclease with regulatory role in breaking down nucleic acids, and genome-wide genetic variants have been associated with SLE, systemic sclerosis and RA (52). *PLD4*-deficient mice develop a severe inflammatory phenotype emphasizing the importance of this gene in immune regulation (54). Functional studies of JDM B cell populations and identified gene pathways in a disease-specific context as well as assessment of the clonality and antigen specificity of B-cell receptors will be important next steps in determining the role of transitional B cells in JDM and relationship to the myositis-specific autoantibodies observed in JDM.

There are important limitations to consider when interpreting the results of this study. While this study is strengthened by the inclusion of JDM treatment-naïve samples with longitudinal time points, we did not have healthy controls samples to be able to determine the specificity of the cell-specific signatures and cell types we identified to JDM. Rather, we are only able to relate our findings to JDM disease activity. Furthermore, due to the rarity of this disease, our sample size was limited, and this did influence our ability to test the association between cell type composition and disease activity with adequate power. One patient, was also treated with B cell depletion, which could skew the composition of cells present. Larger studies to test the association between the number of circulating naïve B cells and CD16^+^ monocytes, or, perhaps, the ratio of these cell types with disease activity, will be important to test in larger cohorts, and additional alterations in immune cell type composition may emerge. Our analysis was also limited to the measurement of 50 pre-specified cell epitopes, which does not fully reflect the compendium of cell surface antibodies, though is the largest number of cell epitopes measured simultaneously to date in JDM. Lastly, JDM is a heterogeneous disease, so it is also possible that certain clinical phenotypes are associated with distinct cell immunophenotypes or subtle signaling pathways not detectable in small studies. Certainly, larger single cell studies considering disease heterogeneity, such as MSA subtype, will be needed in the future to understand if there is an immunologic correlate to explain the disparate clinical phenotypes observed in JDM.

Our study demonstrates the strengths of these experimental and analytic methods to provide biological insights into the immunology of autoimmune diseases as well as evidence and data to inform future immunologic studies. This work is the first comprehensive multi-modal single-cell analysis of the peripheral blood compartment in JDM, and the data will be made available for other myositis researchers to investigate genes and proteins of interest in a cell-specific manner. We hope these findings and accompanying data will lead to many rich insights and hypothesis generation for future JDM research and ultimately help to inform precision medicine approaches to disease management to improve patient outcomes.

## Data Availability Statement

The datasets presented in this study can be found at NCBI Gene Expression Omnibus. The names of the repository/repositories and accession number(s) can be found below: https://www.ncbi.nlm.nih.gov/geo/, GSE190684. The code used for this analysis will be published on Github at “jessicaneely/jdm_citeseq”.

## Ethics Statement

The studies involving human participants were reviewed and approved by the UCSF Institutional Review Board. Written informed consent to participate in this study was provided by the participant or the participants’ legal guardian/next of kin depending on the age of the participant.

## Author Contributions

JN designed the research study, enrolled subjects and performed clinical phenotyping, analyzed data, and wrote the manuscript. GH planned and conducted experiments, analyzed data, and wrote the manuscript. DB analyzed data and wrote the manuscript. YS planned and conducted experiments and revised the manuscript. DL planned and conducted experiments and revised the manuscript. SK designed the research study, enrolled subjects and performed clinical phenotyping, and revised the manuscript. CJY designed the research study, analyzed data, and revised the manuscript. MS designed the research study, analyzed data, and revised the manuscript. All authors contributed to the article and approved the submitted version.

## Funding

This research was supported by the following funding sources granted to JN: Grant 2019124 from the Doris Duke Charitable Foundation, grants from the Cure JM Foundation, a grant from PREMIER, a NIH/NIAMS P30 Center for the Advancement of Precision Medicine in Rheumatology at UCSF (P30AR070155​), and a CARRA Arthritis Foundation grant.

## Conflict of Interest

CJY was employed by Chan Zuckerberg Biohub. CJY is a Scientific Advisory Board member for and holds equity in Related Sciences and ImmunAI, a consultant for and holds equity in Maze Therapeutics, and a consultant for TReX Bio. CJY has received research support from Chan Zuckerberg Initiative, Chan Zuckerberg Biohub, and Genentech.

The remaining authors declare that the research was conducted in the absence of any commercial or financial relationships that could be construed as a potential conflict of interest.

## Publisher’s Note

All claims expressed in this article are solely those of the authors and do not necessarily represent those of their affiliated organizations, or those of the publisher, the editors and the reviewers. Any product that may be evaluated in this article, or claim that may be made by its manufacturer, is not guaranteed or endorsed by the publisher.

## References

[1] Miller FW Lamb JA Schmidt J Nagaraju K. Risk Factors and Disease Mechanisms in Myositis. Nat Rev Rheumatol (2018) 14:255–68. doi: 10.1038/nrrheum.2018.48 PMC6745704 29674613

[2] Mathiesen P Hegaard H Herlin T Zak M Pedersen FK Nielsen S. Long-Term Outcome in Patients With Juvenile Dermatomyositis: A Cross-Sectional Follow-Up Study. *Scand* J Rheumatol (2012) 41(1):50–8. doi: 10.3109/03009742.2011.608376 22044089

[3] Baechler E Bauer J Slattery CA Ortmann WA Espe KJ Novitzke J. An Interferon Signature in the Peripheral Blood of Dermatomyositis Patients Is Associated With Disease Activity. Mol Med (2007) 13:1. doi: 10.2119/2006-00085.Baechler 17515957 PMC1869622

[4] Wong D Kea B Pesich R Higgs BW Zhu W Brown P. Interferon and Biologic Signatures in Dermatomyositis Skin: Specificity and Heterogeneity Across Diseases. PloS One (2012) 7:e29161. doi: 10.1371/journal.pone.0029161 22235269 PMC3250414

[5] Greenberg SA Pinkus JL Pinkus GS Burleson T Sanoudou D Tawil R. Interferon-α/β-Mediated Innate Immune Mechanisms in Dermatomyositis. Ann Neurol (2005) 57:664–78. doi: 10.1002/ana.20464 15852401

[6] Neely J Rychkov D Paranjpe M Waterfield M Kim S Sirota M.. Gene Expression Meta-Analysis Reveals Concordance in Gene Activation, Pathway, and Cell-Type Enrichment in Dermatomyositis Target Tissues. ACR Open Rheumatol (2019) 1(10):acr2.1108. doi: 10.1002/acr2.11081 PMC6917332 31872188

[7] Greenberg SA Higgs BW Morehouse C Walsh RJ Won Kong S Brohawn P. Relationship Between Disease Activity and Type 1 Interferon- and Other Cytokine-Inducible Gene Expression in Blood in Dermatomyositis and Polymyositis. Genes Immun (2012) 13:207–13. doi: 10.1038/gene.2011.61 21881594

[8] Król P Kryštůfková O Polanská M Mann H Klein M Beran O. Serum Levels of Interferon α Do Not Correlate With Disease Activity in Patients With Dermatomyositis/Polymyositis. Ann Rheumatol Dis (2011) 70:879–80. doi: 10.1136/ard.2010.141051 21068097

[9] Mostafavi S Yoshida H Moodley D LeBoité H Rothamel K Raj T. Parsing the Interferon Transcriptional Network and Its Disease Associations. Cell (2016) 164:564–78. doi: 10.1016/j.cell.2015.12.032 PMC4743492 26824662

[10] Rider LG Shah M Mamyrova G Huber AM Rice MM Targoff IN. The Myositis Autoantibody Phenotypes of the Juvenile Idiopathic Inflammatory Myopathies. Med (United States) (2013) 92:223–43. doi: 10.1097/MD.0b013e31829d08f9 PMC3721421 23877355

[11] Fasano S Gordon P Hajji R Loyo E Isenberg DA. Rituximab in the Treatment of Inflammatory Myopathies: A Review. Rheumatology (2017) 56:26–36. doi: 10.1093/rheumatology/kew146 27121778

[12] Piper CJM Wilkinson MGL Deakin CT Otto GW Dowle S Duurland CL. CD19+CD24hiCD38hi B Cells Are Expanded in Juvenile Dermatomyositis and Exhibit a Pro-Inflammatory Phenotype After Activation Through Toll-Like Receptor 7 and Interferon-α. Front Immunol (2018) 9:22. doi: 10.3389/fimmu.2018.01372 29988398 PMC6024011

[13] Throm AA Alinger JB Pingel JT Daugherty AL Pachman LM French AR. Dysregulated NK Cell Plcγ2 Signaling and Activity in Juvenile Dermatomyositis. JCI Insight (2018) 3:e123236. doi: 10.1172/jci.insight.123236 PMC6302943 30429375

[14] Fasth AER Dastmalchi M Rahbar A Salomonsson S Pandya JM Lindroos E. T Cell Infiltrates in the Muscles of Patients With Dermatomyositis and Polymyositis Are Dominated by CD28null T Cells. J Immunol (2009) 183:4792–9. doi: 10.4049/jimmunol.0803688 19752224

[15] Vercoulen Y Bellutti Enders F Meerding J Plantinga M Elst EF Varsani H. Increased Presence of FOXP3+ Regulatory T Cells in Inflamed Muscle of Patients With Active Juvenile Dermatomyositis Compared to Peripheral Blood. PloS One (2014) 9:e105353. doi: 10.1371/journal.pone.0105353 25157414 PMC4144849

[16] Caproni M Torchia D Cardinali C Volpi W Del Bianco E D’Agata A. Infiltrating Cells, Related Cytokines and Chemokine Receptors in Lesional Skin of Patients With Dermatomyositis. Br J Dermatol (2004) 151:784–91. doi: 10.1111/j.1365-2133.2004.06144.x 15491417

[17] Wenzel J Schmidt R Proelss J Zahn S Bieber T Tuting T.. Type I Interferon-Associated Skin Recruitment of CXCR3+ Lymphocytes in Dermatomyositis. Clin Exp Dermatol (2006) 31:576–82. doi: 10.1111/j.1365-2230.2006.02150.x 16716166

[18] Morita R Schmitt N Bentebibel S-E Ranganathan R Bourdery L Zurawski G. Human Blood CXCR5(+)CD4(+) T Cells Are Counterparts of T Follicular Cells and Contain Specific Subsets That Differentially Support Antibody Secretion. Immunity (2011) 34:108–21. doi: 10.1016/j.immuni.2010.12.012 PMC3046815 21215658

[19] Bohan A Peter JB. Polymyositis and Dermatomyositis. N Engl J Med (2010) 292:344–7. doi: 10.1056/NEJM197502132920706 1090839

[20] Mccann LJ Pilkington CA Huber AM Ravelli A Appelbe D Kirkham JJ. Clinical and Epidemiological Research Development of a Consensus Core Dataset in Juvenile Dermatomyositis for Clinical Use to Inform Research. Ann Rheum Dis (2017) 0:1–10. doi: 10.1136/annrheumdis-2017-212141 PMC5816738 29084729

[21] Lazarevic D Pistorio A Palmisani E Miettunen P Ravelli A Pilkington C. The PRINTO Criteria for Clinically Inactive Disease in Juvenile Dermatomyositis. Ann Rheumatol Dis (2013) 72:686–93. doi: 10.1136/annrheumdis-2012-201483 PMC5040631 22736096

[22] Zarinsefat A Hartoularos G Rychkov D Rashmi P Chandran S Vincenti F. Single-Cell RNA Sequencing of Tocilizumab-Treated Peripheral Blood Mononuclear Cells as an *In Vitro* Model of Inflammation. Front Genet (2020) 11. doi: 10.1101/2020.09.11.281782 PMC7813999 33469465

[23] Gayoso A Shor J. JonathanShor/DoubletDetection: doubletdetection v3.0 (v3.0). Zenodo (2020). doi: 10.5281/ZENODO.4359992.

[24] Wolf FA Angerer P Theis FJ. SCANPY : Large-Scale Single-Cell Gene Expression Data Analysis. Genome Biol (2018) 19(1):15. doi: 10.1186/s13059-017-1382-0 29409532 PMC5802054

[25] Behdenna A Haziza J Azencott C-A Nordor A. Pycombat, a Python Tool for Batch Effects Correction in High-Throughput Molecular Data Using Empirical Bayes Methods. bioRxiv, 2020.03.17.995431 doi: 10.1101/2020.03.17.995431 PMC10701943 38057718

[26] Merah-Mourah F Cohen SO Charron D Mooney N Haziot A. Identification of Novel Human Monocyte Subsets and Evidence for Phenotypic Groups Defined by Interindividual Variations of Expression of Adhesion Molecules. Sci Rep (2020) 10:1–16. doi: 10.1038/s41598-020-61022-1 32157175 PMC7064612

[27] Piasecka B Duffy D Urrutia A Quach H Patin E Posseme C. Distinctive Roles of Age, Sex, and Genetics in Shaping Transcriptional Variation of Human Immune Responses to Microbial Challenges. Proc Natl Acad Sci USA (2018) 115:E488–97. doi: 10.1073/pnas.1714765115 PMC5776984 29282317

[28] McInnes L Healy J Saul N Großberger L. UMAP: Uniform Manifold Approximation and Projection. J Open Source Software (2018) 3:861. doi: 10.21105/joss.00861

[29] Traag VA Waltman L Eck NJ. From Louvain to Leiden: Guaranteeing Well-Connected Communities. Sci Rep (2019) 9:1–12. doi: 10.1038/s41598-019-41695-z 30914743 PMC6435756

[30] Bunis DG Andrews J Fragiadakis GK Burt TD Sirota M. dittoSeq: Universal User-Friendly Single-Cell and Bulk RNA Sequencing Visualization Toolkit. Bioinformatics (2020) 36(22-23):5535–6. doi: 10.1093/bioinformatics/btaa1011 PMC8016464 33313640

[31] Love MI Huber W Anders S. Moderated Estimation of Fold Change and Dispersion for RNA-Seq Data With Deseq2. Genome Biol (2014) 15:550. doi: 10.1186/s13059-014-0550-8 25516281 PMC4302049

[32] Wickham H. Ggplot2 Elegant Graphics for Data Analysis Second Edition. Available at: http://www.springer.com/series/6991 [Accessed July 6, 2021].

[33] Yu G Wang L-G Han Y He Q-Y. Clusterprofiler: An R Package for Comparing Biological Themes Among Gene Clusters. Omi A J Integr Biol (2012) 16:284–7. doi: 10.1089/omi.2011.0118 PMC3339379 22455463

[34] Sims GP Ettinger R Shirota Y Yarboro CH Illei GG Lipsky PE.. Identification and Characterization of Circulating Human Transitional B Cells. Blood (2005) 105:4390–8. doi: 10.1182/blood-2004-11-4284 PMC1895038 15701725

[35] Palanichamy A Barnard J Zheng B Owen T Quach T Wei C. Novel Human Transitional B Cell Populations Revealed by B Cell Depletion Therapy. J Immunol (2009) 182:5982–93. doi: 10.4049/jimmunol.0801859 PMC2746373 19414749

[36] Kim H de Jesus AA Brooks SR Liu Y Huang Y VanTries R. Development of a Validated Interferon Score Using NanoString Technology. J Interferon Cytokine Res (2018) 38:171–85. doi: 10.1089/jir.2017.0127 PMC5963606 29638206

[37] Huard C Gullà SV Bennett DV Coyle AJ Vleugels RA Greenberg SA. Correlation of Cutaneous Disease Activity With Type 1 Interferon Gene Signature and Interferon β in Dermatomyositis. Br J Dermatol (2017) 176:1224–30. doi: 10.1111/bjd.15006 27564228

[38] Patel J Maddukuri S Li Y Bax C Werth VP. Highly Multiplexed Mass Cytometry Identifies the Immunophenotype in the Skin of Dermatomyositis. J Invest Dermatol (2021) 141(9):2151–60. doi: 10.1016/j.jid.2021.02.748 PMC8384654 33766508

[39] Narasimhan PB Marcovecchio P Hamers AAJ Hedrick CC. Nonclassical Monocytes in Health and Disease. Annu Rev Immunol (2019) 37:439–56. doi: 10.1146/annurev-immunol-042617-053119 31026415

[40] Randolph GJ Sanchez-Schmitz G Liebman RM Schäkel K. The CD16^+^ (Fcγriii+) Subset of Human Monocytes Preferentially Becomes Migratory Dendritic Cells in a Model Tissue Setting. J Exp Med (2002) 196:517–27. doi: 10.1084/jem.20011608 PMC2196052 12186843

[41] Yang J Zhang L Yu C Yang X-F Wang H. Monocyte and Macrophage Differentiation: Circulation Inflammatory Monocyte as Biomarker for Inflammatory Diseases. Biomark Res (2014) 2:1–9. doi: 10.1186/2050-7771-2-1 24398220 PMC3892095

[42] Guilliams M Bruhns P Saeys Y Hammad H Lambrecht BN. The Function of Fcγ Receptors in Dendritic Cells and Macrophages. Nat Rev Immunol (2014) 14:94–108. doi: 10.1038/nri3582 24445665

[43] Mukherjee R Kanti Barman P Kumar Thatoi P Tripathy R Kumar Das B Ravindran B. Non-Classical Monocytes Display Inflammatory Features: Validation in Sepsis and Systemic Lupus Erythematous. Sci Rep (2015) 5:1–14. doi: 10.1038/srep13886 PMC4566081 26358827

[44] Nehar-Belaid D Hong S Marches R Chen G Bolisetty M Baisch J. Mapping Systemic Lupus Erythematosus Heterogeneity at the Single-Cell Level. Nat Immunol (2020) 21:1094–106. doi: 10.1038/s41590-020-0743-0 PMC7442743 32747814

[45] Arazi A Rao DA Berthier CC Davidson A Liu Y Hoover PJ. The Immune Cell Landscape in Kidneys of Patients With Lupus Nephritis. Nat Immunol (2019) 20:902–14. doi: 10.1038/s41590-019-0398-x PMC6726437 31209404

[46] Torres-Ruiz J Carrillo-Vazquez DA Padilla-Ortiz DM Vazquez-Rodriguez R Nuñez-Alvarez C Juarez-Vega G. TLR Expression in Peripheral Monocyte Subsets of Patients With Idiopathic Inflammatory Myopathies: Association With Clinical and Immunological Features. J Transl Med (2020) 18:1–12. doi: 10.1186/s12967-020-02290-3 32164729 PMC7066841

[47] Sallum AME Kiss MHB Silva CAA Wakamatsu A Vianna MAAG Sachetti S. Difference in Adhesion Molecule Expression (ICAM-1 and VCAM-1) in Juvenile and Adult Dermatomyositis, Polymyositis and Inclusion Body Myositis. Autoimmun Rev (2006) 5:93–100. doi: 10.1016/j.autrev.2005.05.008 16431335

[48] Pinto-Mariz F Rodrigues Carvalho L Prufer DeQueirozCamposAraujo A De Mello W Gonçalves Ribeiro M Cunha MDCSA. CD49d is a Disease Progression Biomarker and a Potential Target for Immunotherapy in Duchenne Muscular Dystrophy. Skelet Muscle (2015) 5:1–10. doi: 10.1186/s13395-015-0066-2 26664665 PMC4674917

[49] Sanz I Wei C Jenks SA Cashman KS Tipton C Woodruff MC. Challenges and Opportunities for Consistent Classification of Human B Cell and Plasma Cell Populations. Front Immunol (2019) 10:2458. doi: 10.3389/fimmu.2019.02458 31681331 PMC6813733

[50] Lipsky PE Lee J Kuchen S Fischer R Chang S. Pre-Naive B Cell Population + Human CD5 Identification and Characterization of a. J Immunol Ref (2021) 182:4116–26. doi: 10.4049/jimmunol.0803391 19299709

[51] Zacca ER Amezcua Vesely MC Ferrero P V. Acosta CDV Ponce NE Bossio SN. B Cells From Patients With Rheumatoid Arthritis Show Conserved CD39-Mediated Regulatory Function and Increased CD39 Expression After Positive Response to Therapy. J Mol Biol (2021) 433:166687. doi: 10.1016/j.jmb.2020.10.021 33098857 PMC9376888

[52] Vuerich M Harshe RP Robson SC Longhi MS. Dysregulation of Adenosinergic Signaling in Systemic and Organ-Specific Autoimmunity. Int J Mol Sci (2019) 20:528. doi: 10.3390/ijms20030528 PMC6386992 30691212

[53] Teodorovic LS Riccardi C Torres RM Pelanda R. Murine B Cell Development and Antibody Responses to Model Antigens Are Not Impaired in the Absence of the TNF Receptor GITR. PloS One (2012) 7:e31632. doi: 10.1371/journal.pone.0031632 22328941 PMC3273462

[54] Gavin AL Huang D Huber C Mårtensson A Tardif V Skog PD. PLD3 and PLD4 are Single-Stranded Acid Exonucleases That Regulate Endosomal Nucleic-Acid Sensing. Nat Immunol (2018) 19(9):942–53. doi: 10.1038/s41590-018-0179-y PMC6105523 30111894

